# Supplementary material for: Hybrid Models and Biological Model Reduction with PyDSTool
Source: PLoS Comput Biol. 2012 Aug 9;8(8):e1002628. doi: 10.1371/journal.pcbi.1002628 (PMC3415397; doi:10.1371/journal.pcbi.1002628)
Supplement: Text S4 — Complete source code for the PyDSTool package (version 0.88.120504). Includes API documentation and help files linking to web pages. This file is identical to the current public release on Sourceforge.net. (ZIP) [file pcbi.1002628.s004.zip › PyDSTool/html/PyDSTool.PyCont.Continuation.LimitCycleCurve-class.html]

xml version="1.0" encoding="ascii"?


PyDSTool.PyCont.Continuation.LimitCycleCurve


| Home | Trees | Indices | Help | PyDSTool |
| --- | --- | --- | --- | --- |

Package PyDSTool ::
Package PyCont ::
Module Continuation ::
Class LimitCycleCurve

[hide private]

[frames] | no frames]

# Class LimitCycleCurve

source code

```
  object --+    
           |    
Continuation --+
               |
              LimitCycleCurve
```

---

```
Wrapper for auto limit cycle computations.

Reimplement these Continuation methods:
    _compute
    _curveToPointset
```


|  |  |
| --- | --- |
| Instance Methods | [hide private] |

|  |  |
| --- | --- |
| \_\_init\_\_(self, model, gen, automod, plot, args=None)  x.\_\_init\_\_(...) initializes x; see x.\_\_class\_\_.\_\_doc\_\_ for signature | source code |

|  |  |
| --- | --- |
| reset(self, args=None)  Resets curve by setting default parameters and deleting solution curve. | source code |

|  |  |
| --- | --- |
| update(self, args)  Update parameters for LimitCycleCurve. | source code |

|  |  |
| --- | --- |
| plot\_cycles(self, coords=None, cycles=None, exclude=None, figure=None, axes=None, normalized=False, tlim=None, method=None, color\_method=`'``default``'`, \*\*plot\_args) | source code |

|  |  |
| --- | --- |
| \_savePointInfo(self, ds=None, evals=None, jacx=None, jacp=None, flow=None, diag=None)  Created a function for this since it needs to be called both in \_compute and when a bifurcation point is found. | source code |

|  |  |
| --- | --- |
| \_compute(self, x0=None, v0=None, direc=1)  NOTE: This doesn't use v0!!!! It gets v0 from c0, which is found from x0. | source code |

|  |  |
| --- | --- |
| info(self) | source code |

**Inherited from `Continuation`**:
`__copy__`,
`__deepcopy__`,
`__repr__`,
`__str__`,
`backward`,
`cleanLabels`,
`computeEigen`,
`display`,
`exportGeomview`,
`forward`,
`getSpecialPoint`,
`testdomain`,
`testdomaingrid`,
`testdomaintangrid`

**Inherited from `Continuation`** (private):
`_Keller`,
`_MoorePenrose`,
`_Natural`,
`_checkForBifPoints`,
`_createTestFuncs`,
`_curveToPointset`,
`_preTestFunc`,
`_system`,
`_systemjac`,
`_systemjac_withpars`,
`_systemjacuser`,
`_systemuser`

**Inherited from `object`**:
`__delattr__`,
`__getattribute__`,
`__hash__`,
`__new__`,
`__reduce__`,
`__reduce_ex__`,
`__setattr__`


|  |  |
| --- | --- |
| Properties | [hide private] |

**Inherited from `object`**:
`__class__`


|  |  |
| --- | --- |
| Method Details | [hide private] |

|  |  |
| --- | --- |
| \_\_init\_\_(self, model, gen, automod, plot, args=None)  *(Constructor)* | source code |

x.\_\_init\_\_(...) initializes x; see x.\_\_class\_\_.\_\_doc\_\_ for
signature

Overrides: object.\_\_init\_\_: *(inherited documentation)*

|  |  |
| --- | --- |
| reset(self, args=None) | source code |

Resets curve by setting default parameters and deleting solution
curve.

Overrides: Continuation.reset

|  |  |
| --- | --- |
| update(self, args) | source code |

Update parameters for LimitCycleCurve.

Overrides: Continuation.update

|  |  |
| --- | --- |
| \_savePointInfo(self, ds=None, evals=None, jacx=None, jacp=None, flow=None, diag=None) | source code |

Created a function for this since it needs to be called both in
\_compute and when a bifurcation point is found. It will have conditional
statements for saving of Jacobian and eigenvalues, as well as other
possible tidbits of information.

Overrides: Continuation.\_savePointInfo

|  |  |
| --- | --- |
| \_compute(self, x0=None, v0=None, direc=1) | source code |

NOTE: This doesn't use v0!!!! It gets v0 from c0, which is found from
x0. This makes sense since there can be no v0 without a c0 (v0 doesn't
make sense for a Hopf point).

Overrides: Continuation.\_compute

|  |  |
| --- | --- |
| info(self) | source code |

Overrides: Continuation.info

  


| Home | Trees | Indices | Help | PyDSTool |
| --- | --- | --- | --- | --- |

|  |  |
| --- | --- |
| Generated by Epydoc 3.0.1 on Fri May 4 15:24:07 2012 | http://epydoc.sourceforge.net |
